# Incidence of hypotension according to the discontinuation order of vasopressors in the management of septic shock: a prospective randomized trial (DOVSS)

**DOI:** 10.1186/s13054-018-2034-9

**Published:** 2018-05-21

**Authors:** Kyeongman Jeon, Jae-Uk Song, Chi Ryang Chung, Jeong Hoon Yang, Gee Young Suh

**Affiliations:** 1 Department of Critical Care Medicine, Samsung Medical Center, Sungkyunkwan University School of Medicine, Seoul, Republic of Korea; 2 Division of Pulmonary and Critical Care Medicine, Department of Medicine, and Critical Care Medicine, Samsung Medical Center, Sungkyunkwan University School of Medicine, 81 Irwon-ro, Gangnam-gu, Seoul, 06351 Republic of Korea; 3 Division of Pulmonary and Critical Care Medicine, Department of Internal Medicine, Kangbuk Samsung Hospital, Sungkyunkwan University School of Medicine, Seoul, South Korea; 4 Division of Cardiology, Department of Medicine, Samsung Medical Center, Sungkyunkwan University School of Medicine, Seoul, Republic of Korea

**Keywords:** Septic shock, Vasopressin, Norepinephrine, Hypotension, Treatment outcome

## Abstract

**Background:**

Vasopressin (AVP) is commonly added to norepinephrine (NE) to reverse shock in patients with sepsis. However, there are no data to support the appropriate strategy of vasopressor tapering in patients on concomitant NE and AVP who are recovering from septic shock. Therefore, the objective of this study was to evaluate the incidence of hypotension while tapering vasopressors in patients on concomitant NE and AVP recovering from septic shock.

**Methods:**

Patients with septic shock receiving concomitant NE and AVP were randomly assigned to taper NE first (NE group) or AVP first (AVP group). The primary end point was the incidence of hypotension within one hour of tapering of the first vasopressor. We also evaluated the association between serum copeptin levels and the occurrence of hypotension.

**Results:**

The study was stopped early due to a significant difference in the incidence of hypotension after 38 and 40 patients were enrolled in the NE group and the AVP group, respectively. There were 26 patients (68.4%) in the NE group versus 9 patients (22.5%) in the AVP group who developed hypotension after tapering the first vasopressor (*p* < 0.001). There was a similar finding during the subsequent tapering of the second vasopressor (64.5% in the NE vs 25.0% in the AVP group, *p* = 0.020). Finally, NE tapering was significantly associated with hypotension during the study period (hazard ratio, 2.221; 95% confidence interval, 1.106–4.460; *p* = 0.025). The serum copeptin level was lower in patients in whom hypotension developed during tapering of AVP than it was in those without hypotension.

**Conclusions:**

Tapering NE rather than AVP may be associated with a higher incidence of hypotension in patients recovering from septic shock who are on concomitant NE and AVP. However, further studies with larger sample sizes are required to better determine the appropriate strategy for vasopressor tapering.

**Trial registration:**

ClinicalTrials.gov, NCT01493102. Registered on 15 December 2011.

## Background

Septic shock is characterized by hypovolemia and decreased vascular resistance, with or without myocardial dysfunction [1]. Therefore, administration of intravenous fluids and catecholamines is critical in patients with septic shock to achieve hemodynamic stability and adequate perfusion to vital organs [2]. However, high doses of norepinephrine (NE) often fail to reverse shock, and vasopressin (AVP) can be added with the intent of either raising the mean arterial pressure (MAP) or decreasing the NE dosage [2]. Vasopressin is effective given its vasoconstrictive action and role in replacing AVP deficiency [3]. Given these characteristics, there is increasing interest in adding AVP early, as an adjunctive agent to NE [4, 5].

As soon as a patient’s hemodynamic variables have stabilized, vasopressor support is gradually tapered in order to decrease the adverse effects of vasopressors [6]. However, clinicians must balance the risks from the potential adverse effects of vasopressors with that of hypotension. Hypotension after discontinuation of NE [7–9] or AVP [9–11] has been reported even after stabilization of septic shock. Such hypotension can cause poor organ perfusion (when the pressure is below an organ’s critical perfusion pressure), and subsequent injury [12]. However, there are few studies that address vasopressor tapering after shock stabilization [9, 13]. Furthermore, the incidence of hypotension after vasopressor tapering is not clearly defined, given the variable study populations and vasopressor titration protocols. In particular, there are no data to support the appropriate strategy of vasopressor tapering when AVP and NE are employed concurrently.

Therefore, we evaluated the incidence of hypotension while tapering vasopressors in patients recovering from septic shock on concomitant norepinephrine (NE) and vasopressin (AVP). We also evaluated the role of serum copeptin in predicting development of hypotension, especially during AVP tapering. AVP tapering was particular important to us because relative AVP deficiency has been hypothesized to contribute to the loss of vascular tone in septic shock [10].

## Methods

The prospective randomized, double-blind, controlled trial on the incidence of hypotension, the Discontinuation Order of Vasopressors in the management of Septic Shock (DOVSS) was conducted at Samsung Medical Center (a 1979-bed, university-affiliated, tertiary referral hospital in Seoul, South Korea) between January 2012 and February 2014. The Institutional Review Board of Samsung Medical Center approved the study protocol. Informed consent was obtained from patients or their legally authorized representative prior to enrollment. This study is registered at ClinicalTrials.gov under the identifier NCT01493102.

All patients who were at least 20 years old, and hospitalized in the medical intensive care unit (ICU) were enrolled if they met all the following inclusion criteria: (1) septic shock with documented site (or strong suspicion) of infection; (2) receiving concomitant NE and AVP infusions; (3) MAP ≥65 mmHg for at least 2 h after reducing NE to 0.3 mcg/kg/min while maintaining AVP of 0.03 U/min. Exclusion criteria were as follows: terminally ill patients classified as “do not resuscitate;” patients who were suspected to have AVP deficiency (e.g. hypothalamic–pituitary–adrenal axis dysfunction, empty sella syndrome); patients with acute myocardial infarction, congestive heart failure or acute mesenteric ischemia; and patients treated with vasopressors other than NE and AVP.

### Initial resuscitation and hemodynamic management of septic shock

A specific protocol for the early recognition and management of patients with severe sepsis or septic shock was implemented at our center in 2004 [14]. In order to improve compliance with the initial resuscitation bundle and management of sepsis, we revised, approved, and promoted our early goal-directed therapy (EGDT) protocol with an educational program named “Emergency Approach to Sepsis Treatment (EAST)” in early 2008 [15]. Our EGDT protocol is an adaptation of the protocol reported by Rivers et al. [16]. Fluid resuscitation and hemodynamic monitoring were initiated in patients fulfilling the criteria for severe sepsis or septic shock, with placement of a central venous catheter via the internal jugular or subclavian vein approach for central venous pressure (CVP) and central venous oxygen saturation (ScvO_2_) monitoring. Broad-spectrum antibiotics were administered as soon as possible.

Hemodynamic resuscitation was conducted according to a predetermined treatment plan. First, isotonic crystalloid was administered in boluses to a target CVP ≥8 mmHg. Second, if systolic blood pressure ≥90 mmHg or mean arterial pressure (MAP) ≥65 mmHg was not achieved with fluid administration, NE was used as a first-line vasopressor to achieve the desired blood pressure. If the target MAP was still not maintained with adequate fluid resuscitation and NE infusion (at 0.3 mcg/kg/min), a supplementary AVP infusion was started at 0.03 U/min. The NE dose was then increased by 0.02mcg/kg/min every 5 min to achieve the target MAP. Finally, ScvO_2_ ≥70% was targeted after CVP and blood pressure goals were met. If ScvO_2_ was < 70% and the hematocrit was < 30%, packed red blood cells were transfused to achieve a hematocrit of at least 30%. If the ScvO_2_ remained < 70% when hematocrit was ≥ 30%, dobutamine was initiated at the treating physician’s discretion and titrated in attempts to reach ScvO_2_ ≥70%. When the patient remained hypotensive after at least one hour of resuscitation with fluids and vasopressors [17], low-dose corticosteroid therapy was recommended as soon as possible after adrenocorticotropic hormone was measured, if possible. However, the time to initiation of low-dose corticosteroid therapy was decided by the treating physician in the emergency department or ICU. Hydrocortisone was administered intravenously every 6 h as a 50-mg bolus for 5 days, and then tapered (50 mg intravenously every 12 h for 3 days, followed by 50 mg intravenously daily for 3 days). Fludrocortisone was not administered in conjunction with hydrocortisone. If hemodynamic stabilization was achieved, the vasopressor was tapered at the discretion of the attending physician, keeping MAP >65 mmHg and urinary output >0.5 mL/kg/h.

### Vasopressor withdrawal protocol and patient assignment

If the target MAP was met and maintained for 2 h with hemodynamic management, NE was titrated to the dose of 0.3mcg/kg/min by 0.02mcg/kg/min every 5 min, keeping MAP >65 mmHg. If the MAP remained stable at > 65 mmHg for another hour, then written informed consent was obtained from the participants. Participants were then randomly assigned to reduce NE first (NE group) or AVP first (AVP group) after another hour, if the target MAP was maintained. A computer-generated randomization list in blocks of four was used for treatment allocation. The randomization procedure and treatment allocation were performed by the research coordinator. The sequence was concealed from patients and investigators until the interventions were completed.

**Fig. 1:**
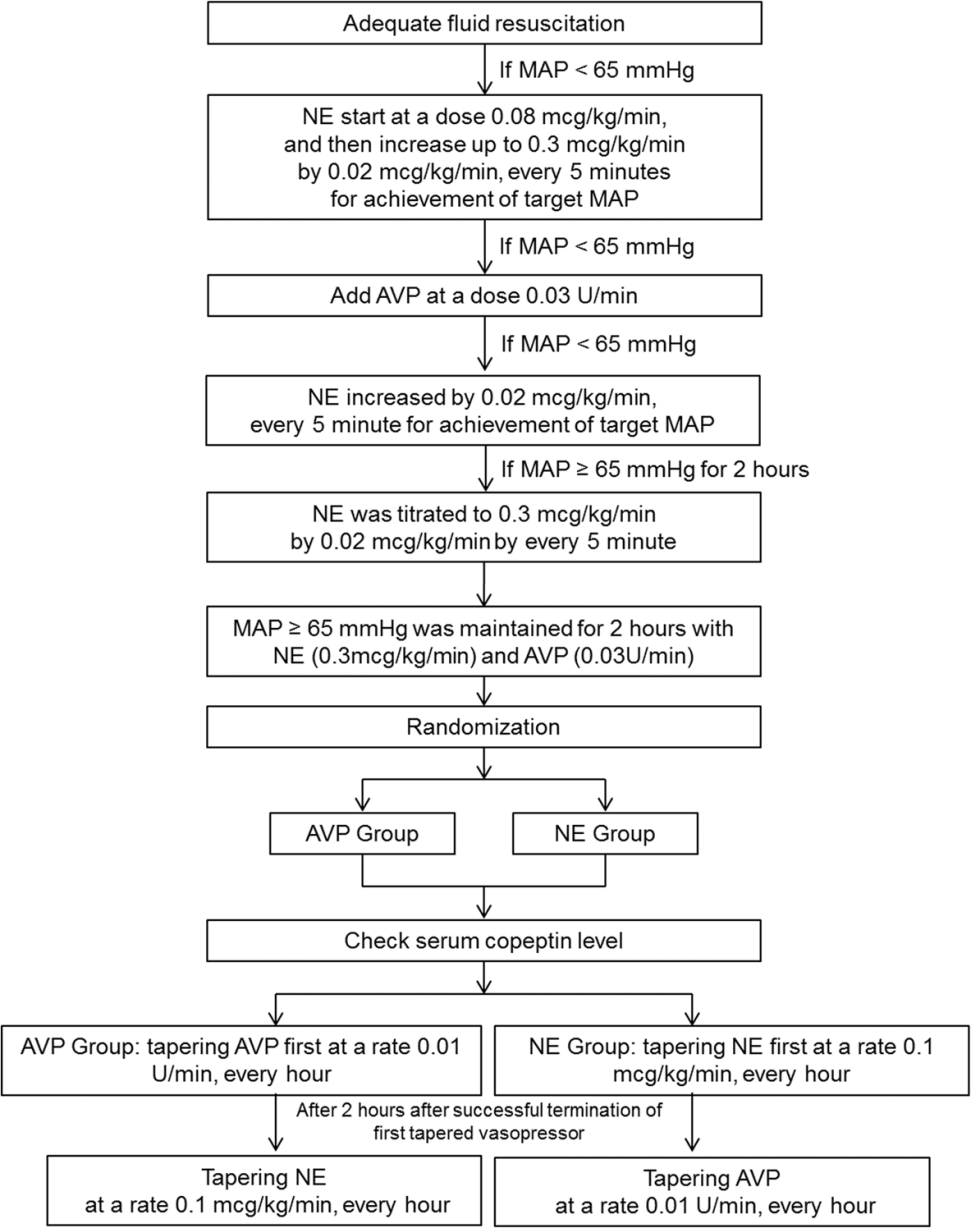
Study protocol on titrating vasopressors. NE, norepinephrine; MAP, mean arterial pressure; AVP, vasopressin

Vasopressors were only tapered when the MAP had been maintained at ≥ 65 mmHg with a constant infusion of both NE (0.3mcg/kg/min) and AVP (0.03 U/min), for at least 2 h. In the NE group, NE was discontinued at the rate 0.1 mcg/kg/min every hour, keeping the AVP infusion at 0.03 U/min; next, AVP was weaned at a rate 0.01 U/min every hour if the MAP was maintained above 65 mmHg for 2 h after successful termination of NE. In the AVP group, AVP was discontinued first and then NE was subsequently withdrawn in the same manner as in the NE group (with the exception of the order). The vasopressor discontinuation continued until the development of hypotension or complete withdrawal of all infused vasopressors. If hypotension developed during vasopressor withdrawal, one or more subsequent interventions were used to maintain the target MAP. These interventions were performed according to the measured CVP. Administration of a fluid challenge (of at least 30 mL/kg of IV crystalloid or equivalent volume of colloid over 30 min) was initially performed to keep CVP >8 mmHg. If CVP was ≥ 8, the discontinued vasopressor was increased up to its dose prior to the hypotension, and then increased according to the protocol (NE, 0.1mcg/kg/min and AVP, 0.0 l unit/min) to maintain the target MAP. If the target MAP was not achieved despite these interventions, the NE dose was increased by 0.1mcg/kg/min every hour until the MAP stabilized. If hypotension developed during weaning of the second vasopressor, the interventions were performed in the same way. The discontinued agent could be restarted if AVP was titrated to the maximum dose of 0.03 U/min or if the required NE infusion exceeded 0.3 mcg/kg/min. Vasopressors were titrated by the bedside nurse and treating physician based on the described study protocol, to maintain the target MAP (Fig. 1).

### Study outcomes

The primary outcome was the incidence of hypotension within one hour after tapering the first vasopressor, which was defined as a sustained decrease in MAP <65 mmHg despite adequate fluid resuscitation. Secondary outcomes included the overall incidence of hypotension during the entire study period, the incidence of hypotension according to the tapering of each vasopressor, ICU mortality, 28-day mortality, and hospital mortality. We also evaluated the association between serum copeptin levels and hypotension during AVP tapering to determine if development of hypotension from AVP tapering is associated with AVP deficiency.

### Measurement of serum copeptin

Measurement of circulating AVP is problematic due to its short half-life, instability, and cumbersome detection methods [18]. In contrast, copeptin is a stable fragment that is located at the C terminal of provasopressin. Copeptin levels directly mirror AVP levels because of its stoichiometric synthesis [19]. Copeptin also exhibits an advantageous biochemical profile for rapid and reliable laboratory testing [19]. Therefore, copeptin has recently been suggested to be a surrogate marker of AVP. Furthermore, one study found that levels of serum copeptin and AVP were strongly correlated in patients with septic shock [20]. Serum copeptin levels were checked in our study using the Copeptin (Human) EIA Kit (CSB-E12130h; CUSABIO CO, Ltd., China), according to the manufacturer’s instructions. The results are expressed in picograms per milliliter. The calibration range was 19.5 pg/mL to 5000 pg/mL, with a detection limit of 19.5 pg/mL. Intra-assay and inter-assay variance was < 8% and 10%, respectively.

### Statistical analysis

**Fig. 2:**
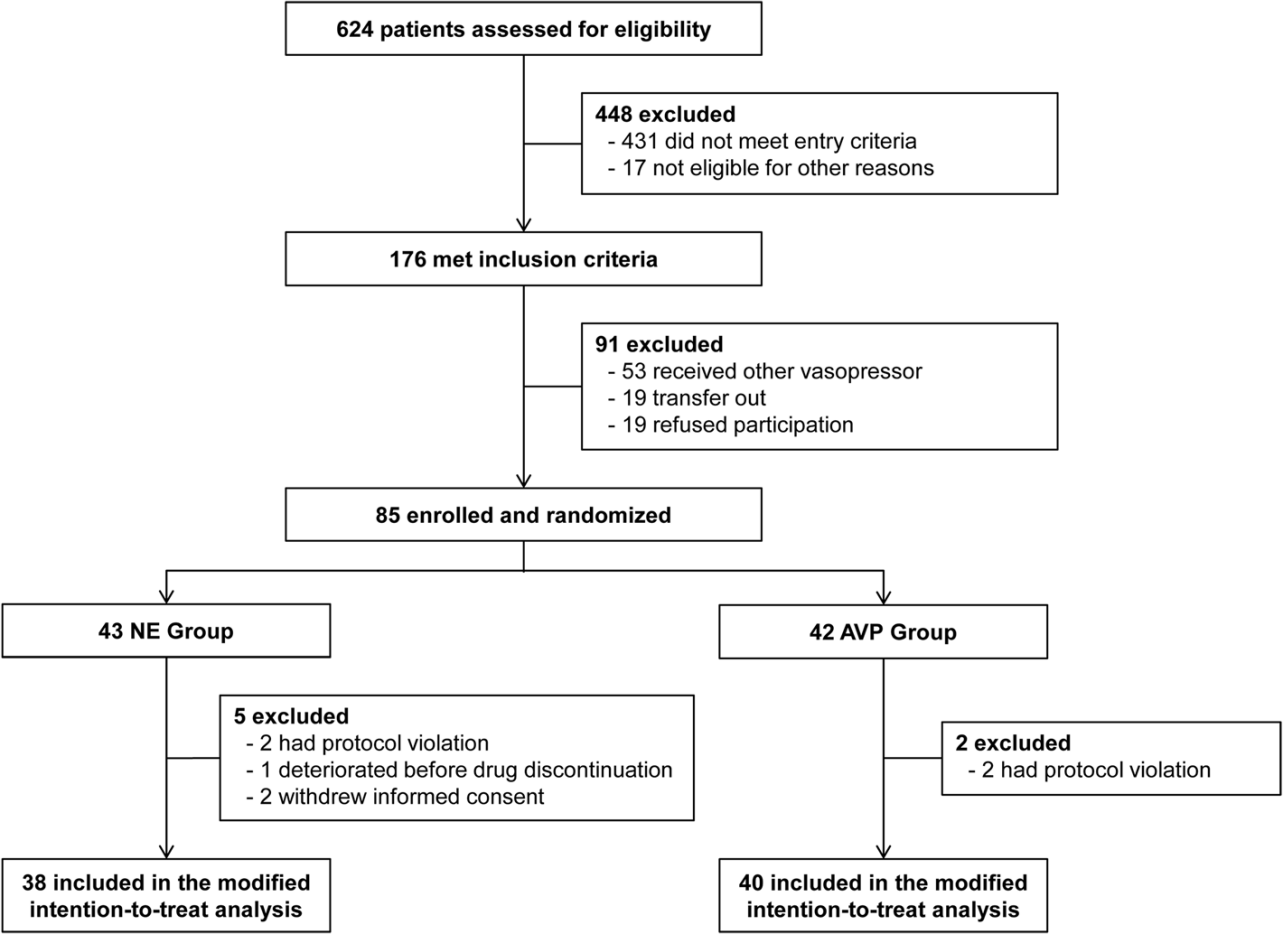
Flow chart of the screening and randomization process

We initially calculated that a sample size of 122 (with 61 per group) was required for enrollment and randomization to detect an absolute difference of 25% in the incidence of hypotension, with two-sided alpha error of 0.05 and power of 80%. This sample size assumed a 55% incidence of hypotension after the initial tapering AVP according to previous study [9]. After considering a dropout rate of 10%, we ultimately needed 134 patients. A preplanned interim analysis was scheduled after enrollment of at least 60% (80 patients) of the planned 134 patients. An O’Brien–Fleming approach was used for sequential stopping rules for safety according to the Lan–DeMets method with an a priori *p* value of 0.025 for stopping [21]. After interim analysis, the study could be stopped, since there was a significant difference in the incidence of hypotension between the two groups. We applied a modified intention-to-treat principle by only analyzing patients who completely followed the study protocol, as this study was designed to evaluate the effect on the incidence of hypotension according to vasopressor tapering (Fig. 2).

Data were compared using the Mann–Whitney U test for continuous variables and the chi-square or Fisher’s exact test for categorical variables. Primary analysis, which compared the incidence of hypotension within one hour of tapering the first vasopressor in the two groups, was evaluated using an unadjusted chi-square test. We also compared clinical parameters, including serum copeptin levels, between the two groups who were classified according to development of hypotension and tapered vasopressor in the subgroups with hypotension development. In order to identify the factors associated with development of hypotension after vasopressor tapering, continuous variables were converted to categorical variables using median cutoff values for multivariable analysis. The chi-square or Fisher’s exact test was used to assess differences between the dichotomous variables. The Cox regression model was used for time-to-event analysis to assess the overall incidence of hypotension during the study period. Time zero for this analysis was defined as the point of tapering the first vasopressor. The variables with a *p* value <0.25 in univariable analysis were entered into a Cox regression model. All tests were two-sided. A *p* value <0.05 was considered statistically significant. Data were analyzed using PASW statistical software version 17 (SPSS, Chicago, IL, USA).

## Results

### Baseline patient characteristics

A total of 624 patients with septic shock who were admitted to the medical ICU were screened for inclusion. After 539 patients were excluded, 85 patients underwent randomization. Of these patients, two withdrew consent after randomization, and four did not have vasopressors tapered according to the study protocol for various reasons. Additionally, one patient clinically decompensated prior to vasopressor tapering. The preplanned interim analysis was ultimately performed in 78 patients, with 38 in the NE group and 40 in the AVP group (Fig. 2). However, serum copeptin levels were unavailable in two patients because of technical errors. After interim analysis, the study was stopped without protocol modification, because there was a significant difference between the two groups in the incidence of hypotension during the first vasopressor tapering.

The baseline characteristics of the enrolled patients are summarized in Table 1. There were 49 men with a median age of 66 years (56–71 years). Pneumonia was the most common cause of septic shock (*n* = 39, 50%). The median sequential organ failure assessment (SOFA) score and simplified acute physiology score 3 (SAPS3) were 10 (8–12) and 74 (63–84), respectively. The median serum copeptin level was 162 pg/mL (107–220) at a median time to randomization of 24.2 h, with no statistically significant difference between the two groups (*p* = 0.640). There was no significant difference between groups in MAP or CVP measured during the study period (Table 1). Patients’ characteristics were well-balanced between the AVP group and NE group, except for arterial partial pressure of oxygen (PaO2)/fraction of inspired oxygen (FiO2) (PF ratio) and need for mechanical ventilation. The patients in the AVP group had lower PF ratios (*p* = 0.014) and received more mechanical ventilation (*p* = 0.009), than did those in the NE group.

**Table 1:** Baseline characteristics of enrolled patients with septic shock

	All patients (*N* = 78)	AVP group (*n* = 40)	NE group (*n* = 38)	*P* value
Age, years	66 (56 – 71)	67 (56 – 73)	64 (55 – 69)	0.206
Gender, male	49 (62.8)	25 (62.5)	24 (63.2)	0.952
Causes of septic shock^a^				0.522
Pneumonia	39 (50.0)	23 (57.5)	16 (42.1)	
Intraabdominal infection	22 (28.2)	10 (25.0)	12 (31.6)	
Urogenital infection	16 (20.5)	7 (17.5)	9 (23.7)	
Cather related infection	5 (6.4)	4 (10.0)	1 (2.6)	
Endocarditis	2 (2.6)	1 (2.5)	1 (2.6)	
Others^b^	3 (3.8)	1 (2.5)	2 (5.3)	
MAP before initial resuscitation, mmHg	52 (45-57)	52 (46-57)	54 (44-60)	0.255
CVP before initial resuscitation, mmHg	7 (4-9	6 (4-8)	7 (5-9)	0.303
MAP at the time of vasopressor initiated, mmHg	55 (51-60)	54 (50-60)	56 (52-60)	0.309
CVP at the time of vasopressor initiated, mmHg	11 (9-14)	10 (8-14)	11 (9-13)	0.954
Total bilirubin, mg/dL	1.05 (0.50-2.30)	1.15 (0.50-2.38)	0.95 (0.50-2.01)	0.802
Serum creatinine, mg/dL	1.34 (0.93-1.99)	1.48 (0.84-1.94)	1.30 (0.98-2.26)	0.960
Lactic acid, mmol/L	3.61 (2.40-5.44)	3.53 (2.41-5.48)	4.11 (2.39-5.65)	0.699
Procalcitonin, ng/mL	12.70 (3.29-37.70)	14.35 (3.52-45.66)	9.91 (2.62-31.42)	0.484
C-reactive protein, mg/mL	13.96 (6.87-24.35)	16.04 (8.29-26.10)	12.99 (6.09-23.17)	0.492
Maximum NE dose during study period, ug/kg/min	0.68 (0.40-1.20)	0.68 (0.40-1.45)	0.68 (0.40-1.03)	0.195
SAPS3	74 (63 – 84)	75 (66 – 92)	72 (61 – 82)	0.192
SOFA score	10 (8 – 12)	10 (8 – 12)	10 (7 – 11)	0.793
Clinical status on randomization				
MAP, mmHg	77 (71 – 81)	75 (68 – 81)	77 (74 – 80)	0.237
CVP, mmHg	10 (8 – 14)	10 (8 – 14)	10 (8 – 14)	0.811
Need for mechanical ventilation	54 (69.2)	33 (82.5)	21 (55.3)	0.009
Need for renal replacement therapy	22 (28.2)	11 (27.5)	11 (28.9)	0.887
Need for dobutamine	6 (7.7)	1 (2.5)	5 (13.2)	0.104
SOFA score	12 (10 – 15)	12 (11 – 15)	12 (9 – 15)	0.413
PF ratio	164.5 (100.2 – 264.1)	132.4 (96.1 – 202.6)	198.9 (133.0 – 290.7)	0.014
Total bilirubin, mg/dL	1.30 (0.68 – 3.70)	1.30 (0.63 – 2.93)	1.35 (0.65 – 4.23)	0.845
Serum creatinine, mg/dL	1.12 (0.72 – 1.95)	1.10 (0.75 – 1.92)	1.23 (0.68 – 1.95)	0.881
Corticosteroid treatment	72 (96.0)	38 (97.4)	34 (94.4)	0.605
Time to randomization	24.2 (13.1 – 44.1)	27.4 (15.1 – 44.4)	19.2 (11.5 – 33.1)	0.108
Total vasopressor duration before tapering first vasoactive agent, hours	24.2 (13.1 – 41.8)	29.0(15.1 – 43.9)	19.2 (11.5 – 33.1)	0.127
Copeptin, pg/mL (*n* = 76)	162 (107 – 220)	148 (100 – 237)	170 (113 – 215)	0.640

Data are presented as frequencies (number of patients), with the percentages in parenthesis, or as medians with interquartile ranges (IQR) in parenthesis

^a^More than one criterion can be used

^b^Others included meningitis (*n* = 1) and deep neck (*n* = 1) and soft tissue infections (*n* = 1)

AVP, vasopressin; CVP, central venous pressure; MAP, mean arterial pressure; NE, norepinephrine; PF ratio, arterial partial pressure of oxygen (PaO2)/fraction of inspired oxygen (FiO2) ratio; SAPS3, simplified acute physiology score 3; SOFA, sequential organ failure assessment

### Incidence of hypotension during vasopressor tapering

There were 26 patients (68.4%) in the NE group versus 9 patients (22.5%) in the AVP group who developed hypotension within one hour after tapering the first vasopressor (*p* < 0.001, Table 2). There was a similar finding during the subsequent tapering of the second vasopressor (64.5% vs 25.0%, *p* = 0.020). Therefore, NE tapering was significantly associated with development of hypotension. However, there were no significant differences in the overall incidence of hypotension during the entire study period between the two groups (Table 2). There were 23 (57.5%) and 13 (34.2%) patients who died during hospitalization in the AVP and NE groups, respectively. Hospital mortality was higher in the patients in the AVP group (Table 2).

**Table 2:** Outcomes by treatment group

	AVP group (*n* = 40)	NE group (*n* = 38)	*P* value
Development of hypotension within one hour after tapering of vasopressor			
Hypotension on tapering the first vasopressor	9 (22.5)	26 (68.4)	< 0.001
Hypotension on tapering sequential second vasopressor (*n* = 43)	20 (64.5)	3 (25.0)	0.020
Hypotension on tapering the first or second vasopressor	29 (72.5)	29 (76.3)	0.700
Time to hypotension after tapering vasopressor, hours (*n* = 58)	4.3(2.5 – 5.1)	2.0 (1.2 – 2.5)	< 0.001
MAP at the time of hypotension developed on tapering of vasopressor, mmHg (*n* = 58)	61 (58 – 62)	62 (59 – 63)	0.111
CVP at the time of hypotension developed on tapering of vasopressor, mmHg (*n* = 58)	10 (7-14)	9 (6-13)	0.810
Total vasopressor duration, hours	58.4(33.9 – 100.0)	43.8 (28.9 – 81.9)	0.169
Clinical outcomes			
ICU mortality	15 (37.5)	11 (28.9)	0.423
ICU length of stay, days	9(6 – 13)	7 (2 – 12)	0.107
28-day mortality	17 (42.5)	12 (32.4)	0.362
Hospital mortality	23 (57.5)	13 (34.2)	0.039
Hospital length of stay, days	25(15 – 38)	21 (13 – 37)	0.542

AVP, vasopressin; NE, norepinephrine; MAP, mean arterial pressure; CVP, central venous pressure; ICU, intensive care unit

### Serum copeptin levels according to group

**Fig. 3:**
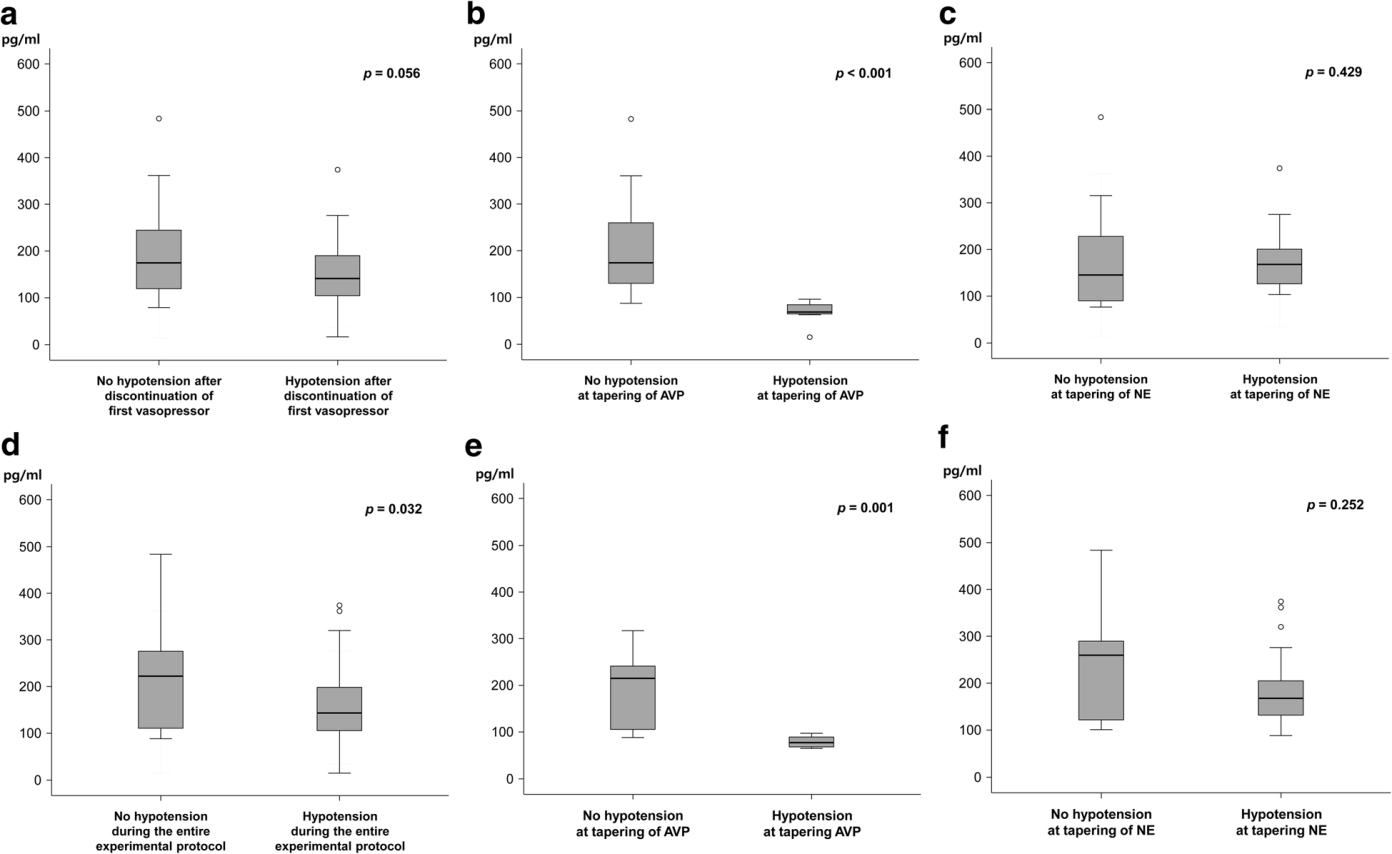
Comparisons of serum copeptin levels between patients with and without hypotension after the first vasopressor was tapered (**a**-**c**) and after sequential tapering all infused vasopressors (**d**-**f**). Data are expressed as medians (interquartile ranges). AVP, vasopressin; NE, norepinephrine

The serum copeptin level was not significantly associated with the order of vasopressor tapering (Fig. 3a). However, the copeptin level was significantly lower in patients who developed hypotension in the AVP group (Fig. 3b), or during the entire experimental period (Fig. 3e). However, these differences were not observed in the NE group (Fig. 3c) or during the entire experimental period (Fig. 3f).

The clinical characteristics were compared between the groups according to which vasopressor was tapered immediately before developing hypotension in the subgroups of patients who developed hypotension (Table 3). The only significant difference was observed in the serum copeptin levels, which were much lower in patients who developed hypotension during AVP tapering than in those who developed hypotension during NE (*p* < 0.001).

**Table 3:** Univariable comparisons of clinical characteristics in patients with hypotension according to the vasopressor tapered immediately before developing hypotension

Variables	Hypotension on tapering of AVP (*n* = 12)	Hypotension on tapering of NE (*n* = 46)	*P* value
Age	67 (59 – 69)	65 (56 – 73)	0.773
Gender, male	10 (83.3)	30 (65.2)	0.307
Causes of septic shock^a^			0.246
Pneumonia	8 (66.7)	20 (43.5)	
Intraabdominal infection	2 (16.7)	14 (30.4)	
Urogenital infection	3 (25.0)	6 (13.0)	
Cather related infection	0 (0.0)	4 (8.7)	
Endocarditis	0 (0.0)	2 (4.3)	
Others^b^	0 (0.0)	3 (6.5)	
MAP before initial resuscitation, mmHg	53 (47 – 57)	51 (42 – 57)	0.214
CVP before initial resuscitation, mmHg	7 (3 – 10)	6 (6 – 8)	0.161
MAP at the time of vasopressor initiated, mmHg	55 (47 – 57)	55 (52 – 60)	0.612
CVP at the time of vasopressor initiated, mmHg	11 (10 – 13)	11 (8 – 14)	0.669
Total bilirubin, mg/dL	0.90 (0.35 – 3.05)	1.20 (0.50 – 2.70)	0.382
Serum creatinine, mg/dL	1.47 (0.71 – 2.01)	1.42 (0.97 – 2.32)	0.687
Lactic acid, mmol/L (*n* = 57)	3.27 (1.91 – 4.62)	4.31 (2.67 – 6.32)	0.229
Procalcitonin, ng/mL (*n* = 53)	13.09 (4.18 – 81.53)	11.01 (2.61 – 30.97)	0.602
C-reactive protein, mg/mL	14.87 (8.51 – 29.72)	12.57 (5.59 – 19.42)	0.129
Maximum NE dose during study period, ug/kg/min	0.74 (0.54 – 1.56)	0.70 (0.46 – 1.20)	0.448
SAPS3	74 (62 – 86)	73 (62 – 84)	0.931
SOFA	9 (8 – 13)	10 (8 – 12)	0.601
Clinical status on randomization			
MAP, mmHg	72 (70 – 78)	77 (73 – 81)	0.138
CVP, mmHg	10 (8 – 12)	10 (8 – 14)	0.420
Need for mechanical ventilation	10 (83.3)	31 (67.4)	0.478
Need for renal replacement therapy	4 (33.3)	14 (30.4)	1.000
Need for dobutamine	1 (8.3)	3 (6.5)	1.000
SOFA	13(11 – 16)	12 (9 – 15)	0.255
PF ratio	118.8 (81.9 – 177.9)	186.9 (105.4 – 278.0)	0.110
Total bilirubin, mg/dL	1.35 (0.53 – 3.68)	1.40 (0.80 – 3.88)	0.744
Serum creatinine, mg/dL	1.25 (0.60 – 1.99)	1.26 (0.81 – 2.21)	0.508
Corticosteroid treatment	10 (90.9)	42 (95.5)	0.495
Copeptin, pg/mL (*n* = 56)	77 (67 – 90)	168 (131 – 207)	< 0.001
Time to randomization, hours	25.1 (14.1 – 41.8)	21.1 (11.8 – 46.2)	0.818
Time to discontinuation of vasopressors just before hypotension developed	28.8 (18.5-44.3)	22.0 (13.6-47.2)	0.946
Time to hypotension after discontinuation of vasopressor, hours	2.5 (1.1 – 3.3)	2.5 (1.9 – 4.7)	0.442
MAP at the time of hypotension developed on tapering of vasopressor, mmHg	61 (57 – 63)	62 (59 – 63)	0.214
CVP at the time of hypotension developed on tapering of vasopressor, mmHg	10 (6 – 12)	10 (7 – 14)	0.735
Total vasopressor duration, hours	63.4 (38.9 – 122.6)	57.8 (38.9 – 88.0)	0.578
Clinical outcomes			
ICU mortality	5 (41.7)	13 (28.3)	0.486
ICU length of stay, days	12 (8 – 22)	8 (3 – 12)	0.108
28-day mortality	5 (41.7)	17 (37.8)	1.000
Hospital mortality	6 (50.0)	20 (46.5)	0.686
Hospital stay, days	27 (19 – 30)	22 (14 – 39)	0.617

Data are presented as frequencies (number of patients), with the percentage in parenthesis, or as medians with interquartile ranges (IQR) in parenthesis

^a^More than one criterion can be used

^b^Others included meningitis (*n* = 1) and deep neck (*n* = 1) and soft tissue infections (*n* = 1)

AVP, vasopressin; CVP, central venous pressure; MAP, mean arterial pressure; NE, norepinephrine; PF ratio, arterial partial pressure of oxygen (PaO2)/fraction of inspired oxygen (FiO2) ratio; SAPS3, simplified acute physiology score 3; SOFA, sequential organ failure assessment

### Clinical factors associated with hypotension

Univariable comparisons of clinical variables were performed to identify factors associated with hypotension after vasopressor tapering (Table 4). Interestingly, low serum C-reactive protein (CRP) was associated with the development of hypotension. In addition, following vasopressor tapering the median serum copeptin level was lower in patients who developed hypotension (144 pg/mL, IQR 105–199 pg/mL) than it was in those who did not (223 pg/mL, IQR 109–281 pg/mL, *p* = 0.032). NE tapering was significantly associated with hypotension (*p* = 0.035). However, there were no significant differences between the two groups with regard to mortality or length of stay. In a multivariable analysis using a Cox proportional hazards model, hypotension was only significantly associated with NE tapering during the entire experimental period (adjusted hazard ratio, 2.221; 95% confidence interval, 1.106–4.460; *p* = 0.025).

**Table 4:** Univariable comparisons of clinical characteristics between patients with hypotension and without hypotension after sequential tapering all vasopressors

Parameter	Hypotension (*n* = 58)	No hypotension (*n* = 20)	*P* value
Age	65 (57 – 72)	66 (54 – 70)	0.828
Gender, male	40 (69.0)	9 (45.0)	0.056
Causes of septic shock^a^			0.588
Pneumonia	28 (48.3)	11 (55.0)	
Intraabdominal infection	16 (27.6)	8 (40.0)	
Urogenital infection	9 (15.5)	5 (25.0)	
Cather related infection	4 (6.9)	1 (5.0)	
Endocarditis	2 (3.4)	0 (0.0)	
Others^b^	3 (5.2)	0 (0.0)	
MAP before initial resuscitation, mmHg	52 (43 – 57)	52 (50 – 58)	0.414
CVP before initial resuscitation, mmHg	6 (4 – 8)	7 (5 – 10)	0.250
MAP at the time of vasopressor initiated, mmHg	55 (51 – 60)	54 (50 – 59)	0.649
CVP at the time of vasopressor initiated, mmHg	11 (9 -14)	10 (8 – 13)	0.516
Total bilirubin, mg/dL	1.15 (0.50 – 2.70)	0.90 (0.60 – 1.83)	0.406
Serum creatinine, mg/dL	1.42 (0.95 – 2.09)	1.16 (0.76 – 1.86)	0.351
Lactic acid, mmol/L	4.19 (2.64 – 6.07)	3.15 (2.36 – 4.99)	0.329
PCT, ng/mL	11.81 (2.62 – 34.12)	15.36 (3.61 – 54.96)	0.417
CRP, mg/mL	12.60 (5.83 – 21.19)	20.66 (11.97 – 26.10)	0.026
Maximum NE dose during study period, ug/kg/min	0.70 (0.50 – 1.31)	0.40 (0.31 – 1.12)	0.020
SAPS3	73 (62 – 74)	79 (72 – 90)	0.297
SOFA score	10 (8 – 12)	10 (8 – 11)	0.936
Clinical status on randomization			
MAP, mmHg	77 (71 – 80)	76 (68 – 83)	0.936
CVP, mmHg	10 (8 – 14)	10 (9 – 13)	0.606
Need for mechanical ventilation	41 (70.7)	13 (65.0)	0.635
Need for renal replacement therapy	18 (31.0)	4 (20.0)	0.344
Need for dobutamine	4 (6.9)	2 (10.0)	0.643
SOFA score	12 (10 – 15)	12 (9 – 15)	0.704
PF ratio	164.9 (99.1 – 267.9)	164.5 (103.1– 258.5)	0.972
Total bilirubin, mg/dL	1.40 (0.78 – 3.70)	1.25 (0.50 – 3.73)	0.453
Serum creatinine, mg/dL	1.26 (0.74 – 2.10)	1.01 (0.68 – 1.26)	0.093
Corticosteroid treatment	52 (94.5)	20 (100.0)	0.500
Copeptin, pg/mL (*n* = 76)	144 (105 – 199)	223 (109 – 281)	0.032
Time to randomization, hours	23.3 (12.1 – 44.7)	24.4 (14.8 – 30.5)	0.868
MAP at the evaluation of outcomes, mmHg	62 (59 – 63)	72 (67 – 77)	< 0.001
CVP at the evaluation of outcomes, mmHg	10 (7 – 14)	10 (8 – 14)	0.499
NE tapering at the evaluation of event outcomes	46 (79.3)	11 (55.0)	0.035
AVP tapering at the evaluation of event outcomes	12 (20.7)	9 (45.0)	0.035
Total vasopressor duration, hours	57.8 (39.0 – 110.6)	30.8 (21.3 – 36.7)	< 0.001
Clinical outcomes			
ICU mortality	18 (31.0)	8 (40.0)	0.463
ICU length of stay, days	9 (4 – 13)	7 (3 – 12)	0.387
28-day mortality	22 (38.6)	7 (35.0)	0.775
Hospital mortality	26 (44.8)	10 (50.0)	0.689
Hospital length of stay, days	23 (14 – 37)	19 (14 – 42)	0.936

Data are presented as frequencies (number of patients), with the percentage in parenthesis, or as medians with interquartile ranges (IQR) in parenthesis

^a^More than one criterion can be used

^b^Others included meningitis (*n* = 1) and deep neck (*n* = 1) and soft tissue infections (*n* = 1)

AVP, vasopressin; CVP, central venous pressure; MAP, mean arterial pressure; NE, norepinephrine; PF ratio, arterial partial pressure of oxygen (PaO2)/fraction of inspired oxygen (FiO2) ratio; SAPS3, simplified acute physiology score 3; SOFA, sequential organ failure assessment

## Discussion

In this randomized controlled trial on the incidence of hypotension with vasopressor tapering, we found that hypotension developed more commonly in patients in whom NE was tapered first. In a Cox proportional hazards model, NE tapering was significantly associated with hypotension. In patients in whom hypotension developed during AVP tapering, however, the serum copeptin level was significantly lower than it was in those without hypotension.

There are guidelines on vasopressor initiation in the hemodynamic management of patients with septic shock [2]. However, there are no guidelines addressing the safe tapering of these medications in patients receiving AVP in addition to NE. Some physicians favor reducing NE first, because the incidence and duration of AVP deficiency is unclear due to variable causes of septic shock and disease courses [22, 23]. In contrast, others suggest that AVP should be tapered first, because NE is easier to titrate than is AVP, as AVP significantly affects the cardiac output, splanchnic system, and balance between oxygen delivery and consumption [24]. In one retrospective cohort study, tapering of AVP before NE resulted in a greater incidence of clinically significant hypotension than did the reverse order [9]. In a more recent retrospective cohort study of 154 patients with septic shock, patients in whom AVP was tapered first developed hypotension that required intervention more commonly than did those in whom NE was tapered first [13]. In contrast to the findings from these retrospective observational studies [9, 13], however, the current prospective randomized controlled study revealed that NE tapering was more likely to lead to hypotension than AVP tapering during the entire experimental period (79.3% vs 55.0%, *p* = 0.035). It is difficult to explain this result; however, it might be explained by the different time to tapering vasopressors. In a prospective cohort study, AVP deficiency was mainly observed ≥36 h after shock onset [22]. In addition, interaction between AVP and corticosteroid treatment should be considered [25, 26]. In the previous study [9] patients having NE discontinued first were more commonly treated with corticosteroids than those having AVP discontinued first. In this study, however, there was no difference in the dose and duration of corticosteroid infusion between the two groups. Difference in half-life between NE and AVP could affect our result. The longer effective half-life of AVP (10–20 min) than NE (2–2.5 min) may help avoid rebound hypotension after discontinuation of the drug.

Although multiple pathophysiologic mechanisms are responsible for cardiovascular failure in patients with septic shock [1], inadequate plasma concentrations of AVP prevent the restoration of normal vascular tone [1, 27]. Relative AVP deficiency has been reported in one third of patients with septic shock [22]. Theoretically, therefore, exogenous AVP administration could restore hemodynamic variables in septic shock that is poorly responsive to standard catecholamine therapies [28]. However, the exact onset time and frequency of AVP deficiency were not clearly determined [22, 23]. Another question in this study sought to determine the association between AVP deficiency and the development of hypotension during AVP tapering. The incidence of hypotension during AVP tapering was 15% at the median time to AVP tapering of 29 h. In addition, the serum levels of copeptin, the sensitive surrogate marker of AVP release [29], were significantly lower in these patients compared to those who did not develop hypotension. These results are comparable to those of previous studies, which indicated that the incidence of relative AVP deficiency was 15–22% approximately 24–36 h after shock onset [22, 23]. However, the significant difference in serum copeptin was not observed in cases in which hypotension developed during NE tapering or in those without hypotension. Therefore, these finding suggest that the serum copeptin level is a useful surrogate marker to select patients who are more sensitive to exogenous AVP [30].

Our study has several limitations that should be mentioned. First, our study was conducted at a single center, which limits the generalization of our findings to other institutions or populations with different resources [31]. In the future, large multi-center trials could substantiate our findings. Second, patients in whom AVP was tapered first had lower PF ratios and therefore a greater need for mechanical ventilation, than did those in the NE group. This discrepancy may have been associated with more patients with pneumonia in the AVP group, which may have facilitated AVP secretion by hypoxemia [32], and could have an effect on our results. However, the serum copeptin level (directly mirroring AVP levels) at randomization was not significantly different between the two groups. Third, we did not have further information on myocardial dysfunction and its influence on our results. Therefore, further studies using advanced hemodynamic monitoring including cardiac index would be needed. Finally, this study was sufficiently powered to detect a difference in the primary outcome, but not clinically important secondary outcomes, such as mortality and length of stay in the ICU.

## Conclusion

The incidence of hypotension was high during vasopressor tapering, which was related to the vasopressor itself but not to the order of vasopressor tapering. Given the tentative results from our study, further studies with larger sample sizes are required to better determine the appropriate strategy for vasopressor tapering. However, NE tapering was significantly associated with hypotension developed during vasopressor tapering. Therefore, our results suggest that tapering AVP before NE (rather than the reverse) may lead to a lower incidence of hypotension in patients recovering from septic shock who are on concomitant AVP and NE.

## Abbreviations

AVP: Vasopressin
CVP: Central venous pressure
EGDT: Early goal directed therapy
ICU: Intensive care unit
MAP: Mean arterial pressure
NE: Norepinephrine
PF ratio: Arterial partial pressure of oxygen (PaO2)/fraction of inspired oxygen (FiO2) ratio
SAPS3: Simplified acute physiology score 3
ScvO_2_: Central venous oxygen saturation
SOFA: Sequential organ failure assessment
